# Evaluation of Colour Changes in Nanocomposite-Based Bulk-Fill and Universal Composite Using Different Polishing Systems

**DOI:** 10.3290/j.ohpd.b5740315

**Published:** 2024-09-12

**Authors:** Ezgi Eroğlu Çakmakoğlu, Metin Bakir

**Affiliations:** a Pediatric Dentist, Department of Pediatric Dentistry, Faculty of Dentistry, Bingol University, Bingol, 12000 Turkey. Idea, hypothesis, experimental design, performed the experiments in partial fulfilment of requirements for a degree, wrote the manuscript, proofread the manuscript, performed a certain test, consulted on and performed statistical evaluation, and contributed substantially to discussion.; b Restorative Dentist, Department of Restorative Dentistry, Faculty of Dentistry, Bingol University, Bingol, 12000 Turkey. Idea, hypothesis, experimental design, performed the experiments in partial fulfilment of requirements for a degree, wrote the manuscript, proofread the manuscript, performed a certain test, consulted on and performed statistical evaluation, contributed substantially to discussion.

**Keywords:** bulk-fill composite, colour changes, polishing systems, universal composite

## Abstract

**Purpose:**

Resins composites are widely used in modern dentistry because of their aesthetic and physical properties. However, discoloration of anterior tooth restorations is a common complaint. Understanding the factors affecting the colour stability of resin composites can lead to longer-lasting repairs. This study aimed to evaluate and compare the colour changes of nanocomposite-based bulk-fill and universal resin composites after immersion in coffee using various polishing systems.

**Materials and Methods:**

A total of 160 samples were prepared using four different composite groups, with 40 pieces for each combined group. Based on the finishing procedure, the samples were divided into four subgroups for each composite group. Three different polishing procedures were applied to the samples according to the manufacturer’s instructions. The control group was not subjected to any treatment. Initial colour measurements were performed using a VITA Easyshade V spectrophotometer. After the initial measurements, the samples were immersed in a Nescafe coffee solution for seven days, followed by colour measurements. Data were analysed using the Kolmogorov–Smirnov test and two-way analysis of variance. Tukey’s honest significant difference (HSD) test was used to determine differences between subgroups.

**Results:**

The results indicate that bulk-fill resins exhibit more discolouration than universal composites; however, this difference was not statistically significant. The resin group with the smallest discolouration was Ceram X, and the most effective polishing method was Twist polishing.

**Conclusion:**

Final surface polishing significantly reduced the composites’ discolouration. These findings support the selection of appropriate materials and polishing techniques to achieve aesthetic outcomes and colour stability in dental restorations.

Currently, composites with highly advanced properties, in which inorganic and organic materials are used together, are preferred as dental filling materials.^24,33^ With increasing interest in dental aesthetics, resin composites have been developed as an alternative to amalgam, and their use as the primary material in dental restorations has become widespread. Using resin composites as permanent restorative materials not only preserves healthy tooth tissue by minimising tooth tissue loss but also provides a natural appearance.^22^ The quest to achieve optimum aesthetic results by enhancing this natural appearance has led to innovations in tooth-coloured materials and their application techniques. As a result, changes made in the formulations of resin composites have improved their physical and mechanical properties, improved their aesthetic appearance, and ensured their safe use in both anterior and posterior teeth.^7,8,28,37^

The physical and mechanical properties of the materials used in dentistry directly affect the success of restorations. The popularity of light-cured composites can be attributed to their biocompatibility, abrasion resistance, and, most importantly, the natural appearance they offer.^18^ Despite these advantages, the increasing application of light-cured composites requires technical precision and prolongs clinical procedures. To ensure optimum adaptation to the cavity and light penetration, the layers in the layering technique should not exceed 2 mm and be applied obliquely or horizontally. Additional disadvantages of layering techniques include interlayer contamination, bonding inadequacies, and difficulties in placing small gaps.^38^ To overcome these disadvantages, manufacturers have recently developed bulk-fill resin composites that can be applied in a single 4–5 mm mass.^14,16^ When bulk-fill composites are compared to the traditional resin composites used with the layered technique, it has been reported that they reduce the cap mobility during polymerisation and show good performance in marginal integrity.^29^

Another critical issue for aesthetic restorative materials is that they can comprehensively imitate the natural appearance of teeth, which is directly related to the materials’ colour harmony and colour stability.^12,13^ Predicting and preventing colour changes by understanding composite materials physical and chemical effects can extend the restorations’ life. Various intrinsic and extrinsic factors affect the colour stability of resin composites.^15^ One of these factors is the finishing and polishing procedures.^27^ Various methods are available for finishing and polishing resin composite restorations.^3^ Most studies have indicated that aluminium oxide polishing systems are the gold standard for microfilled resin composites. However, diamond particle polishing systems, which have been reported to significantly affect hybrid resin composites polishing, also have an essential place.^22^ Currently, silicon carbide and diamond particle polishing systems are among the systems preferred by most clinicians. These systems may require one or more processes and can vary significantly in the composition and hardness of the abrasive particles they contain.^32^ Recent developments have produced one-step polishing and polishing discs with pure diamond particles.^4^ According to the literature review, it has been observed that high gloss surfaces are obtained with aluminium oxide polishing systems in microfilled composites. However, in nano-filled resin composites, brighter surfaces are obtained with diamond polishing systems.^2^

The main reasons for replacing composite restorations are unpredictable colour stability and susceptibility to staining.^6,36^ This study aims to fill the knowledge gap by providing more information on the colour stability and optical properties of bulk-fill composites. The findings will contribute significantly to the understanding of colour changes in dental composite materials and will compare the colour changes of nanofiller-based bulk-fill and universal resin composites after exposure to coffee using different polishing systems.

The hypotheses of this research are as follows:

Our study’s null hypothesis (H0) is that the resin material and polishing method will not affect coloration.

## MATERIALS AND METHODS

Manufacturer information and compositions of the tested resin composites are presented in Table 1, and the polishing systems are presented in Table 2. We used A2 colour for all resin composites. Teflon moulds with a diameter of 10 mm and depth of 2 mm were used to prepare the composite samples. The resin composites were placed inside Teflon moulds. Strip tape and glass were placed under and on top of the Teflon moulds during the sample preparation. A total of 160 samples were prepared, with 40 samples in each of the four resin composite groups. Each prepared sample was polymerised for 20 s on both the top and bottom surfaces using an LED light device (WoodPacker LED). G, China), emitting light in the 420–480 nm range.

**Table 1:** Resin composites used

Material	Resin type	Manufacturer	Composition according to manufacturer’s information
Ceram X	Nanoceramic Universal resin composite	Dentsply Sirona, Switzerland	Bis-GMA, TEGDMA, UDMA. Methacrylate-modified polysiloxane, dimethacrylate resin, fluorescent pigment, UV stabiliser, camphoroquinone, ethyl-4 (dimethylamino) benzoate, titanium oxide pigments aluminium silicate pigments Barium-aluminium-borosilicate glass (1.1–1.5 µm); methacrylate silicon dioxide nanofiller (10 nm), 76% by weight, 57% by volume
Tetric EvoCeram	Nanohybrid Universal resin composite	Ivoclar Vivadent, Schaan, Liechtenstein	Dimethacrylate comonomers (Bis-GMA, Bis-EMA, UDMA), photoinitiator/polymerisation enhancers (Camphoroquinone, TPO, dibenzoyl germanium by-product-Ivocerin) Barium aluminium silicate glass, YbF3, spherical mixed oxide fillers (62.5% by weight) and prepolymers (wt. 17%, 60% by volume), particle size 1 μm
Filtek Ultimate	Nanoceramic bulk-fill composite	3M ESPE, Seefeld, Germany	Bis-GMA, UDMA, Ba-AlSi glass, prepolymer fillers (monomer, glass filler and ytterbium fluoride), spherical mixed oxide. 78.5/63.3% weight to volume ratio, filler particles 0.013.5 μm filler particle size
SonicFill 2 Bulk Fill	Nanohybrid bulk-fill composite	Kerr, Orange, CA, USA	Glass, oxide, chemicals (10–30%), 3-trimethoxysilylpropyl methacrylate (10 –30%), silicon dioxide (5–10%), ethoxylated bisphenol A dimethacrylate (1–5%), bisphenol A bis(2-hydroxy -3-methacryloxypropyl) ether (1–5%), and triethylene glycol dimethacrylate (1–5%), 83.5 wt. filler size 30 nm–0.4 μm

**Table 2:** Polishing systems used

Polishing type	Abrasive particle	Producer	Particulate content
OptiOne Step	One-stage polishing system	3M ESPE, St. Paul, MN, USA	3–14 µm aluminium oxide particles, diamond dust and aluminium-oxide-coated tyres
Sof-Lex Disc	Four-stage polishing system Aluminium oxide-coated discs	3M ESPE, St. Paul, MN, USA	Thick (55 µm), medium (40 µm), thin (24 µm), very thin (8 µm)
Twist Dia	Two-stage polishing system	Kuraray, Japan	Diamond-coated flexible silicone spirals, pre-polisher: 14 µm, high-shine polisher: 10 µm

The samples were divided into four subgroups (n = 10) for each composite group during polishing. The samples were polished according to the manufacturer’s instructions. The control group was finished using a strip matrix, without further polishing. All polishing and polymerisation protocols were performed in accordance with the manufacturer’s instructions.

The surfaces of the samples in the rubber group were polished using a single-step rubber-polishing system (OptiOne Step 3M, USA). The samples were polished for 20 s at high pressure and 15,000 rpm and then polished for 20 s with light pressure at 5,000 rpm using the same rubber polisher.

The surfaces of the samples in the Sof-Lex group were polished using a four-step aluminium oxide-coated polishing system (Sof-Lex, 3M ESPE, St. Paul, MN, USA). The samples were polished sequentially from coarse to fine using thick (55 µ), medium (40 µ), fine (24 µ), and ultra-fine (8 µ) polishing discs at 5,000 rpm without water cooling, with each step lasting 20 s.

The Twist group samples were polished using diamond-particle-impregnated polishing spirals (Twist Dia Pre-polisher, Kuraray, Japan). First, a thick-grit (14 µ) spiral rubber (pre-polisher rubber) was applied for 20 s in the opposite direction of the clock at 5,000 rpm without water cooling. Then, the fine-grit (10 µ) spiral rubber (high-shine polisher rubber) was applied for 20 s in the opposite direction of the clock at 2,000 rpm without water cooling. To reduce technical variability, the sole operator performed this step.

The samples prepared for colour measurement were kept in distilled water at 37°C for 24 h prior to measurements. Before each measurement, the samples were rinsed with distilled water and dried using a drying paper. Initial colour values (L0, a0, b0) were measured using a VITA Easyshade V spectrophotometer (VITA Zahnfabrik, Germany). After the initial measurements, the samples were immersed in a coffee solution (Nescafe, Nestle, Switzerland, Batch 91591210 B) for 7 days. The choice of the coffee brand stems from its widespread utilisation in the European market. The coffee, obtained from a traditional departmental establishment, was meticulously prepared using freshly heated tap water, a practice that is renewed every day. Fifteen grams of ground coffee were poured into 500 mL of heated water, subjected to a 10 min infusion, and subsequently underwent a filtration process before being transferred to designated containers. Colour measurements were performed in the same manner. Each colour measurement was conducted under standard conditions in a white room with a white background illuminated only by a Fluorescent Daylight Lamp (Master TL-D 90 Graphica 18W965SLV/10; Philips, Netherlands). The spectrophotometer was calibrated before each measurement. The final CIELAB scale and L1, a1, and b1 values were measured. ΔE was calculated using the following equation:

ΔE = [(L1 - L0)2 + (a1 - a0)2 + (b1 - b0)2] ½.

The CIE L* a* b* values after immersion in the beverages are denoted as L1, a1, and b1, whereas the initially measured CIE L* a* b* values are represented as L0, a0, and b0, respectively.

All samples in the solutions were kept in a drying oven at 37°C between measurements. The solutions were regularly changed at 24-h intervals. Colour measurements were made just before immersion (baseline), after 1 week. Based on previous studies, an acceptable ΔE value of 3.7 was adopted.^9,11^

The SPSS 22.00 software package was used to analyse the data obtained from the measurements. The normality of the data distributions was assessed using the Kolmogorov–Smirnov test. Parametric tests, specifically two-way ANOVA, were used to compare the data with normal distributions. Post-hoc tests, specifically Tukey’s honest significant difference (HSD) test, were applied to assess differences between subgroups.

## RESULTS

In this study, four nanocomposites consisting of two different types of universal and bulk-fill composites were investigated. In terms of average discolouration values, it was observed that the universal composites exhibited less discolouration than the bulk-fill composites, although the difference between them was not statistically significant.

**Fig 1:**
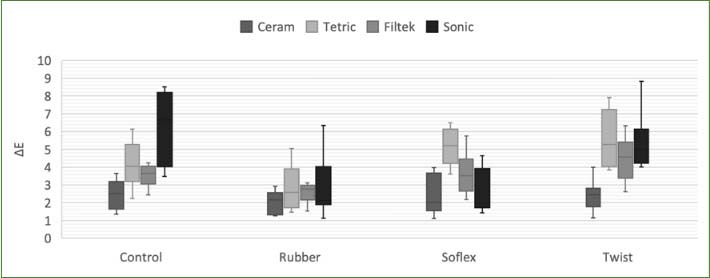
Box-plot showing ΔE, mean and standard deviation values.

The average ΔE values were calculated for different resin composite groups subjected to different polishing systems and then immersed in a coffee solution based on the results of two-way ANOVA. A statistically significant difference was observed between ΔE values (p < 0.05). The average ΔE values determined by immersion of each sample in the coffee solution and the results of the statistical analysis are presented in Table 3. The results are illustrated in the box-plot graphs in Figure 1. Among the resin composites, the highest average ΔE value was observed in the Tetric group (4.41 ± 1.67), while the lowest average value was found in the Ceram X group (2.33 ± 0.84). Among the polishing systems, the highest average ΔE value was measured in the rubber group (4.40 ± 1.22), whereas the lowest average ΔE value was observed in the Twist group (2.56 ± 1.13). When examining the subgroups, the highest average ΔE value was recorded in the control polishing group of the Sonic composite (6.29 ± 2.01), while the lowest value was measured in the Twist group (2.01 ± 0.64).

**Table 3:** Mean colouration values (ΔE) and statistical analysis result

			POLISHING TYPE				TOTAL
			CONTROL	TWİST	SOF-LEX	RUBBER	
MATERİAL	UNIVERSAL	CERAM X	2.46 ± 0.8^Aa^	2.01 ± 0.64^Aa^	2.40 ± 1.09^Aa^	2.43 ± 0.85^Aa^	2.33 ± 0.84^A^
		TETRIC	4.14 ± 1.27^Aab^	2.79 ± 1.26^Aa^	5.16 ± 1.12^Bb^	5.57 ± 1.60^Bb^	4.41 ± 1.67^B^
	BULK-FILL	FILTEK	3.51 ± 0.61^Aab^	2.57 ± 1.22^Aa^	3.62 ± 1.16A^Bab^	4.40 ± 1.22^Bb^	3.52 ± 1.11^B^
		SONIC	6.29 ± 2.01^Ba^	2.86 ± 1.71^Ab^	2.51 ± 1.29^Ab^	5.44 ± 1.60^Ba^	4.28 ± 2.3^B^
TOTAL			4.10 ± 1.88^a^	2.56 ± 1.13^b^	3.42 ± 1.58^ab^	4.46 ± 1.81^a^	

*Different letters indicate statistically significant differences. Uppercase letters compare vertically, and lowercase letters correspond to the horizontal order. **The aggregate values encompassing all samples attributed to the specific material and polishing technique are delineated at the termini of corresponding rows and columns.

In examining ΔE values according to the resin composites variable; no significant differences were found among the groups in the Twist polishing group. In the Sof-Lex group, Tetric resin showed the highest level of discolouration, and this difference was statistically significant. In the Twist polishing group, Ceram X resin showed the lowest discolouration value, and this difference was also statistically significant.

No significant differences were found between the polishing methods in the Ceram X group when the ΔE values were analysed according to the polishing method. However, in the Tetric and Filtek resin groups, the Twist polishing group showed significantly less discolouration. In the Sonic resin group, the Twist and Sof-Lex polishing groups exhibited significantly less discolouration than the Control and Rubber-polishing groups.

Statistical analysis revealed that the Ceram X resin group exhibited the least discolouration, whereas the Twist polishing group had the most effective polishing method. Additionally, the average values of the Twist and Sof-Lex polishing groups and the Ceram X and Filtek resin groups were below the acceptable colour-change threshold (ΔE < 3.7).

The analysis of the total average values of the final polishing groups revealed that the Twist-polishing group was significantly lower than the Control and Rubber-polishing groups. There were no significant differences among the other polishing methods. Ceram X and Tetric groups are Universal, Filtek and Sonic resin groups are bulk-fill resin composites. Total ΔE and standard deviation values were sorted according to the composite types.

Regarding the total average values of the resin composite groups, the ΔE value of Ceram X was significantly lower than those of the other resin composites. No significant differences were found between the polishing methods in the Ceram X group when the ΔE values were analysed according to the polishing method. However, in the Tetric and Filtek resin groups, the Twist polishing group showed significantly less discoloration. In the Sonic resin group, the Twist and Sof-Lex polishing groups exhibited significantly less discoloration than the Control and Rubber groups.

## DISCUSSION

The study results indicate that the null hypothesis (H0) has been rejected. It has been observed that there is a significant difference in colour change after immersion in coffee among different combinations of dental filling materials and polishing procedures. These findings underscore the potential impact of dental filling material and polishing procedure selection on colour stability.

Colour change in restorative materials used for aesthetic purposes is a significant problem. This negatively affects the aesthetic appearance of the material and leads to adverse outcomes in terms of cost and time for both patients and dentists.^15^ The factors contributing to colour change include water absorption, material structure, dynamic changes in the oral environment, inadequate polymerisation, poor oral hygiene, colouring agents in consumed food and beverages, pH, fabrication errors, and insufficient polishing.^5,19,21^

Colour changes in restorations are directly associated with patients’ dietary habits, particularly beverage consumption.^27^ In recent years, the increasing number of coffee shops in China has increased coffee consumption.^1^ In this study, coffee was selected based on its consumption and colouring properties. According to coffee producers, consuming a cup of coffee usually takes an average of 15 min, and a coffee consumer drinks an average of 3.2 cups of coffee per day. Therefore, the 7-day immersion of the coffee samples corresponded to 7 months of coffee consumption.^21^ In this study, the samples were immersed in all beverages for 7 days to determine the colour measurement values to ensure standardisation.

In this study, the traditional nanoceramic composite Ceram X showed a slight colour change among all materials. This can be attributed to its high filler content, low resin volume fraction, and reinforced photopolymerisation system with less camphor quinone (CQ)/amine usage, resulting in better colour stability than other bulk-fill composites.^30^

This study showed that bulk-fill composites are more prone to discolouration than traditional composites. The different optical properties can be attributed to the improved formulations of the bulk-fill composites, including increased photoinitiator content or an additional type of photoinitiator, higher translucency, and different fillers.^5,30^ As previously reported, using blue light-absorbing photosensitisers and amines as initiators in composite formulations can lead to undesired oxidative colour changes.^25,31^

Changes in the filler type to enhance the light transmission depth through refractive index matching between fillers and the organic matrix to achieve higher translucency in bulk-fill composites can result in different optical properties. The monomer system in bulk-fill composites, which allows stress relaxation by forming a cross-linked network structure during polymerisation, is another factor that may affect the optical properties of the composite.^17^

Various surface finishing and polishing procedures can alter the surface roughness of resins, thereby affecting their colour stability.^20,26,27^ Marigo et al^23^ emphasised that the surface gloss depends on the flexibility of the polishing material and the type and hardness of the abrasive particles present in the polishing material. Türkün et al^34^ found that a polishing procedure to obtain a smooth surface in resin composites restorations could increase colour resistance. In another study, the highest surface gloss was achieved with diamond-particle-impregnated polishing material.^35^ In our study, a two-step Twist system, a new-generation spiral-shaped polishing material containing diamond abrasive particles, was used, and the lowest colour change was observed in this polishing group. Similar results have been reported previously.^34,35^

Multistep finishing and polishing systems have been reported to outperform single-step systems and are more effective in achieving surface smoothness.^17^ Flury et al stated that a multistep aluminium oxide-based finishing and polishing system (Sof-Lex, 3M ESPE, USA) created less surface roughness than a two-step diamond particle-based system dental material.^10^ Consistent with these findings, our study identified a higher colour change in the rubber group, representing a single-step polishing system. No significant differences were observed among the polishing methods in the Ceram X group. The Twist polishing technique exhibits a lower average degree of colouration on the Ceram X material, but this difference is not statistically significant. This can be attributed to the superior colouration resistance of the Ceram X material compared to other resins. However, in the Tetric and Filtek resin groups, the Twist polishing group showed significantly less colour change. In the Sonic resin group, the Twist and Sof-Lex polishing groups exhibited significantly less colour change than the Control and Rubber groups.

This study has some limitations. For instance, the sample surfaces were flat, whereas resin composite restorations in the clinical setting had convex and concave surfaces and irregular shapes. Additionally, restoration surfaces in clinics are often prepared using burs before polishing. The staining solution used in this study did not account for all substances to which the composite restorative materials were exposed. Other factors that influence the degree of total colour change include thermal cycling, ageing, food products and beverages, and wear.

## CONCLUSIONS

Colouring beverages such as coffee can cause colour changes in resin composites. The type of resin composite and finishing polishing procedures chosen for the restoration affect colouration. Bulk-fill resin composites exhibited slightly higher colour change than universal composites, but this difference was not statistically significant. The resin group with the least colour change was found to be Ceram X. The two-stage spiral-shaped polishing system (Twist), which contains diamond particles as abrasives, is considered more advantageous and effective than other polishing materials in terms of polishing time and colour change during surface polishing of composite materials. Final surface polishing significantly reduced discoloration of resin composites.
